# Potential Natural Biomolecules Targeting JAK/STAT/SOCS Signaling in the Management of Atopic Dermatitis

**DOI:** 10.3390/molecules27144660

**Published:** 2022-07-21

**Authors:** Spandana Rajendra Kopalli, Venkata Prakash Annamneedi, Sushruta Koppula

**Affiliations:** 1 Department of Bioscience and Biotechnology, Sejong University, Seoul 05006, Korea; spandanak@sejong.ac.kr; 2 Convergence Science Research Center, College of Pharmacy and Institute of Chronic Diseases, Sahmyook University, Seoul 01795, Korea; bobbymelina@gmail.com; 3 Department of Biotechnology, College of Biomedical and Health Science, Konkuk University, Chungju 27381, Korea

**Keywords:** cytokines, atopic dermatitis, interleukins, SOCS proteins, JAK-STAT pathway, natural biomolecules

## Abstract

Atopic dermatitis (AD) is a chronic inflammatory skin disease caused by the dysregulation of cytokines and other immune mediators. JAK/STAT is a classical signal transduction pathway involved in various biological processes, and its dysregulation contributes to the key aspects of AD pathogenesis. Suppressor of cytokine signaling (SOCS) proteins negatively regulate the immune-related inflammatory responses mediated by the JAK/STAT pathway. JAK/STAT-mediated production of cytokines including IL-4, IL-13, IL-31, and TSLP inhibits the expression of important skin barrier proteins and triggers pruritus in AD. The expression of SOCS proteins regulates the JAK-mediated cytokines and facilitates maintaining the skin barrier disruptions seen in AD. STATs are crucial in dendritic-cell-activated Th2 cell differentiation in the skin, releasing inflammatory cytokines, indicating that AD is a Th2-mediated skin disorder. SOCS proteins aid in balancing Th1/Th2 cells and, moreover, regulate the onset and maintenance of Th2-mediated allergic responses by reducing the Th2 cell activation and differentiation. SOCS proteins play a pivotal role in inflammatory cytokine-signaling events that act via the JAK/STAT pathway. Therapies relying on natural products and derived biomolecules have proven beneficial in AD when compared with the synthetic regimen. In this review, we focused on the available literature on the potential natural-product-derived biomolecules targeting JAK/STAT/SOCS signaling, mainly emphasizing the SOCS family of proteins (SOCS1, SOCS3, and SOCS5) acting as negative regulators in modulating JAK/STAT-mediated responses in AD pathogenesis and other inflammatory disorders.

## 1. Introduction

Atopic dermatitis (AD) is a chronic inflammatory skin disease characterized by relapsing eczema accompanied by dry skin and persistent itching [1]. The complexity of chronic AD is increased by the interplay of skin barrier dysfunction and immune dysregulation, resulting in the perpetuation of AD signs and symptoms [2,3]. Skin barrier dysfunction facilitates the entry of allergens and pathogens, which leads to lesions on the surface of the skin, inflammation beneath the skin, and pruritus [4,5]. This ultimately results in epicutaneous sensitization and skin irritability to nonspecific stimuli, eliciting a localized cutaneous inflammatory response [1]. Dysregulation of cytokines and other immune mediators might be one of the key factors involved in the pathogenesis of AD [6,7]. The stromal cells, including the keratinocytes in the disrupted barrier epidermis, release high amounts of thymic stromal lymphopoietin (TSLP), IL-25, and IL-33, which are responsible for promoting T-helper type 2 (Th2)-dominant immune responses via OX40L/OX40 ligand signaling [8,9]. These inflammatory pathways, in turn, suppress the expression of filaggrin (FLG) in keratinocytes and worsen epidermal barrier dysfunction [10,11].

AD is a Th2 cell immune-response-mediated inflammatory skin disease and is considered one of the core pathways leading to cutaneous inflammation. Acute AD skin lesions have a significantly higher number of Th2 cells expressing IL-4 and IL-13 when compared to unaffected AD skin [8]. The severity of AD is well correlated with IL-1/IL-13 related modules such as chemokine ligand (CCL), periostin, and galectin-9 [12,13,14]. Dendritic cells (DCs) activated by IL-4 and IL-13 produce type-2 chemokines (CCL17, CCL18, CCL22, and CCL26), which are chemoattractive to Th2 cells and are overexpressed in AD skin lesions [15,16,17]. In addition, Th2 cells release IL-22, which is linked to the chronicity and amplification of skin inflammation in AD [18,19]. Potential proteins such as FLG and loricrin (LOR) are important in the formation of an effective skin barrier, and Th2-derived cytokines such as IL-4 and IL-13 reduce FLG and LOR expression [20,21,22]. Further, IL-4 and IL-13 also inhibit the cytoplasmic-to-nuclear translocation of OVO-like transcriptional repressor (OVOL)-1, an upstream factor of FLG and LOR expression [23]. Modulation of Th2 response by targeting respective cytokines and their receptors has recently become the primary focus of drug development strategies in the treatment of AD [24,25].

Further, IL-24 produced by IL-1/13 mediates the signal transducer and activator of transcription (STAT)-6. IL-24 expressed in keratinocytes activates the JAK1/tyrosine kinase2/STAT3 pathway and inhibits the expression of FLG [26]. IL-24 plays an important role in skin barrier dysfunction seen in AD. The skin barrier dysfunction also induces TSLP, IL-25, and IL-33, which are part of the type-2 cell-mediated immune deviation [27,28]. Previous research has found that AD skin lesions have higher levels of TSLP, IL-25, and IL-33 [29,30,31]. TSLP increases OX40L expression in DCs and induces IL-4, 5, and 13 from OX40^+^ T-cells [9,32,33]. IL-31, predominantly produced by Th2 cells, is a pruritogen in patients with AD [34]. In AD patients, activated leukocytes expressed higher levels of serum IL-31, which is well correlated with disease severity [16,35,36]. Overexpression of IL-31 potently induces pruritus and promotes elongation and branching of sensory nerve fibers, thereby increasing the intensity of AD symptoms [37]. IL-31 has the ability to stimulate the production of pro-inflammatory cytokines and chemokines by activating the JAK1/JAK2 and STAT3 pathways [34].

The JAK-STAT pathway is involved in the regulation of various body functions and plays a major role in some important biological processes including cell proliferation, differentiation, apoptosis, immune regulation, and homeostasis [38]. JAK/STAT is the classical signal transduction pathway for the production of numerous cytokines and growth factors. STATs are phosphorylated upon cytokine stimulation of JAK, resulting in the dimerization of STAT, followed by translocation of STAT to the nucleus through the nuclear membrane to regulate the expression of their target genes [39]. JAK/STAT signaling is responsible for the transmission of numerous cytokines such as IL-4, IL-2, and IL-7, as well as growth factors such as granulocyte-macrophage colony-stimulating factor (GM-CSF), growth hormone (GH), epidermal growth factor (EGF), platelet-derived growth factor (PDGF), and interferons (IFN) [40]. Further, STATs play an important role in the activation of signals and transduction. The cytoplasmic STATs are the downstream targets of JAKs, which are the most crucial cytokine-activated transcription factors in the process of immune response [39]. STATs play an important role in neuronal and cytokine-mediated signaling pathways such as ILs, IFNs, EPO, PRL, GH, oncostatin M, and ciliary neutrophil factors, with broader biological functions in the treatment of disease resistance [41,42].

Recently, there has been an increasing interest in understanding the role of the JAK/STAT pathway as a key area of focus in providing novel insights into the pathogenesis of AD. Further, negative regulators, consisting of SOCS proteins that modulate the JAK/STAT pathway, are regarded as among the major signaling mechanisms in the management of AD. In the following sections, we attempt to generate an overview of SOCS proteins signaling in the context of JAK/STAT pathway modulation, focusing on AD pathogenesis.

## 2. SOCS Proteins Expression and JAK/STAT Signaling in AD

The SOCS protein family of molecules play an important role as negative regulators of cytokine signaling [43,44,45]. The SOCS protein family consists of eight members, including the cytokine-inducible SH2-containing protein (CIS) and SOCS1-7, which are known to be implicated in multiple immunologic-related pathologies and inflammatory diseases [45]. Cytokines such as ILs and IFNs interact with extracellular ligands through their cell-surface receptors, resulting in the activation of intracellular JAK family kinases (JAK1, JAK2, JAK3, and TYK2) and STAT1-6 [46,47]. SOCS proteins, which have a central SH2 domain and a conserved C-terminus, play an important role in controlling cytokine responses at various inflammatory sites and act as potent inhibitors of the JAK-STAT signaling pathway [48,49].

Previous studies revealed that SOCS1 and SOCS3 are induced by several inflammatory and anti-inflammatory cytokines such as IL-2, IL-4, IL-6, and IFNs and inhibit cytokine actions [50,51,52]. SOCS proteins regulate Th1-Th2 cell balance and reduce Th2-induced inflammation [53]. Modulation of SOCS proteins expression influences various inflammatory cascades at different points such as Th2 differentiation, Th2 cell activation, and Th2 cytokine effects. A significant number of studies have pointed out the importance of SOCS proteins that can modulate the JAK/STAT pathway and are emerging therapeutic targets in the treatment of AD and other inflammation-related pathologies [45,48]. In the following section, we discussed the three major SOCS proteins, namely SOCS1, SOCS3, and SOCS5, involved in the pathophysiology of AD and other inflammatory skin disorders.

### 2.1. SOCS1

As one of the major members of the SOCS family of proteins, SOCS1 is involved in actions ranging from immune modulation to cell cycle regulation [54]. Earlier reports indicated that SOCS1-deficient mice failed to survive, with severe lymphopenia, peripheral T-cell activation, liver degeneration, and macrophage infiltration in several organs [55,56,57]. SOCS1 is a negative regulator of inflammation, strongly induced by its ligand following the stimulation of a toll-like receptor (TLR). SOCS1 suppresses the production of pro-inflammatory cytokines such as IFN-γ, IL-6, and TNF-α [58]. Cells lacking SOCS1 strongly respond to TLRs stimulation [50]. Further, SOCS1 is also involved in the regulation of TLR-induced nuclear factor kappa-B activity (NF-κB) through direct interaction with the p65 subunit [59].

SOCS1 plays an important role in the regulation of IFN-γ-mediated signaling. Accumulation of activated DCs secretes high levels of B-cell-activating factor (BAFF)/BLysS, leading to abnormal B-cell growth and differentiation associated with the generation of auto-reactive B-cells, which results in the rise of chronic inflammatory diseases [60]. The abnormal expression of CD8^+^, CD11c^lo^, CD11b^−^, and B220^−^ in activated DCs leads to highly expressed IFN-γ and BAFF/BLysS in SOCS1-deficient mice [60]. SOCS1 induced by various cytokines acts via JAK-STAT signaling and strongly inhibits this pathway by binding to all JAK family members [61].

SOCS1 is proposed as an important molecule to inhibit Th2 and Th1 cell development by IFN-γ and IL-4, respectively [62,63,64]. SOCS1-deficient T-cells exhibited sustained phosphorylation of STAT4 or STAT6 when stimulated with IL4 or IL-12, respectively, and this subsequently resulted in excessive differentiation of T-cells towards Th2 or Th1 cells. SOCS1 selectively blocks Th2 differentiation via inhibition of IL-4-mediated signaling [65]. In relation to skin disorders such as AD and psoriasis, SOCS1 protein overexpression was observed when compared to normal skin, and this overexpression inhibited the IFN-γ-induced transactivation of a STAT1-binding promoter in keratinocytes, proving that SOCS1 protein is a potential target for the treatment of cytokine-induced inflammatory skin disorders [66].

### 2.2. SOCS3

The SOCS3 gene, cloned in 1997, is one of the major SOCS family proteins that have been shown to possess immunoregulatory roles in inflammatory-associated infections including AD [67,68]. The main function of SOCS3 is to inhibit the signaling induced by IL-6 by preventing JAK-mediated activation of STAT3, and it is also involved in the regulation of NF-κB [69,70]. Cytokine receptor signaling in response to a diverse range of cytokines and growth factors was attenuated by SOCS3 through multifaceted mechanisms [67]. SOCS3 can interact directly with phosphorylated JAKs, thereby inhibiting JAK kinase activation [71]. STAT recruitment is prevented by SOCS3 by binding to phosphotyrosine residues on receptor chains [67]. The receptor-bound STATs are inhibited by SOCS3-linked phosphotyrosine residues in the membrane-proximal region of the GH receptor.

SOCS3 is a gene strongly associated with AD susceptibility, as elevated expression of SOCS3 was observed in the skin of AD patients [72]. SOCS3 expression in T-cells inhibits Th1 development and promotes Th2 responses, increasing the risk for Th2-mediated allergic diseases, including AD [73]. Overexpression of SOCS3 protein impairs IL-12-mediated Th1 differentiation, and this process is reflected in enhanced Th2 development in SOC3 transgenic mice [73,74]. Further, SOCS3 transgenic mice showed elevated levels of Th2 responses, expressing SCOS3 protein constitutively. Decreased Th2 development was observed in the dominant-negative mutant SOCS3 transgenic mice and heterozygous deletion of SOCS3 mice [73]. In view of the above reports, SOCS3 plays a beneficial role in the regulation of Th2-mediated allergic responses, indicating that SOCS3 might be considered a potential target in the treatment and management of various allergic diseases including asthma and AD.

### 2.3. SOCS5

SOCS5, a member of the STAT-induced STAT inhibitor (SSI) protein family, is known to be predominantly expressed in lymphoid-tissue-related organs [75,76]. Earlier reports indicated that SOCS5 is involved in the regulation of T-cell differentiation by attenuating IL-4 signaling [77]. IL-4 is a key cytokine that promotes Th2 development by activation of STAT6 and is selectively impaired in Th1 cells [78]. SOCS5 attaches to the cytoplasmic domain of the IL-4R α-chain in a non-tyrosine-based interaction and structurally interferes with interaction between JAK1 and IL-4R, resulting in the inhibition of IL-4-induced STAT6 activation [78,79,80]. The binding of SOCS5 to IL-4R occurs via the N-terminal region, suggesting that this region regulates target specificity [80,81]. SOCS5 negatively regulates Th2 development and is exclusively expressed in committed Th1 cells. The expression of SOCS5 seems to be regulated by the Th1-driving cytokine IL-12 [80]. The reduction of IL-4-mediated Th2 development in transgenic mouse T-cells expressing SOCS5 constitutively strengthens the fact that SOCS5 is a specific inhibitor of IL-4-dependent signaling in the control of Th2 differentiation [76]. This indicates that the induction of SOCS5 during initial T-cell activation plays a marked role in regulating the direction of Th2-cell differentiation.

Earlier reports showed that SOCS5 can inhibit JAK1 and JAK2 autophosphorylation and serves as a tumor suppressor gene in several malignancies by negatively regulating JAK-STAT signaling and EGF pathways [82,83,84]. Further, experimentally induced SOCS5 expression attenuated the IL-4-mediated STAT6 activation, reducing the DCs’ function in chronic-lymphocytic-leukemia-associated immune suppression [85].

SOCS5 has been shown to be involved in a variety of allergic disease states, including AD and asthma [86,87,88]. Comparable SOCS5 expression was found in patients having Th2-dominant AD. SOCS5 reduces the eosinophil production infiltration in allergic conjunctivitis and balances the Th1/Th2-mediated response, since eosinophil production is stimulated by Th2 cytokines including IL-4. Furthermore, SOCS5 knock-out mice had higher levels of peritoneal IL-2 and IFN cytokines, both of which promote Th1 differentiation [86]. Based on the above literature, SOCS5 expression and its mediation as a negative regulator of JAK/STAT signaling highlights the point that agents acting as agonists in enhancing SOCS5 protein expression might have therapeutic benefits in alleviating allergy-related diseases, including AD.

## 3. Natural Biomolecules Targeting JAK/STAT/SOCS Signaling in AD Management

Increasing evidence supports the involvement of SOCS proteins in coordinating Th1/Th2 cellular profiles and modulation of JAK/STAT signaling in the management of various allergic diseases such as AD [48,89,90]. In particular, SOCS1, SOCS3, and SOCS5 proteins have been shown to strongly act as negative regulators in the modulation of JAK/STAT signaling [82,91]. The multiple effects of SOCS proteins in different in vitro and in vivo experimental models call for thorough investigations to clarify their main mechanisms and targets. In the following section, we discuss reported studies from natural-product-derived biomolecules for their possible role in exhibiting beneficial effects in AD and other related skin disorders, mainly focusing on SOCS proteins expression and JAK/STAT signaling.

### 3.1. Resveratrol

**Figure 1:**
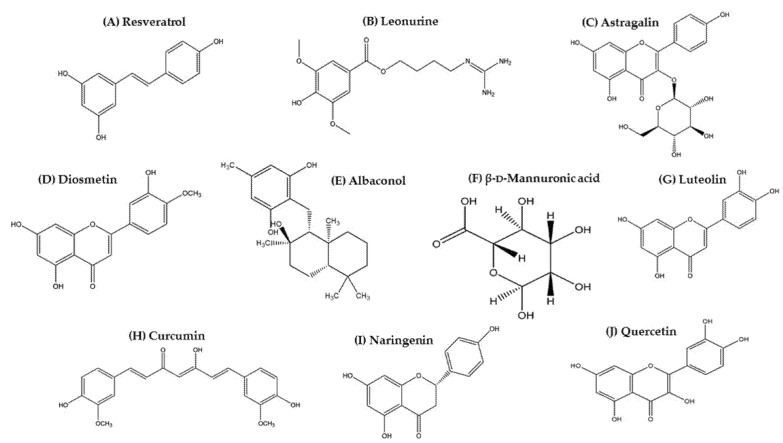
Chemical structures of selected natural compounds targeting SOCS expression and JAK/STAT-signaling pathways.

Resveratrol (3,5,4′-trihydroxy-trans-stilbene, Figure 1A) is a natural bioactive polyphenol and phytoalexin antioxidant found in a wide variety of plant species [92,93,94,95]. Resveratrol was first isolated from the roots of white hellebore (*Veratrum grandiflorum* O. Loes) in 1940 [96] and later from *Polygonum cuspidatum*, which is part of traditional Chinese and Japanese medicine [97]. Resveratrol is one of the major bioactive constituents found in the skin and seeds of over 70 plants including grapes, berries, grains, tea, and peanuts [98,99]. Pharmacologically, resveratrol possesses antitumor, anti-inflammation, anti-oxidant, and cardioprotective effects [100,101,102]. Several studies have stated that resveratrol delays the progression of neurodegenerative disorders and can reduce microglial activation in brain ischemia [103,104,105]. Further, resveratrol aids in various dermatological problems such as acne, exfoliate eczema, psoriasis, UV-mediated skin damage, and other skin pathologies induced by microbial infections [93,106].

In a previous study, resveratrol was reported to upregulate SOCS1 production in LPS-stimulated RAW 264.7 macrophages by inhibiting micro RNAs such as miR155, which act as regulators of inflammatory responses [107]. Further, resveratrol blocked STAT3 signaling by induction of SOCS1, thereby attenuating the STAT3 phosphorylation in squamous cell carcinoma of the head and neck (SCCHN) cells [108]. Resveratrol delayed the onset of skin lesions in AD-like pathologies and improved 2,4-dinitrochlorobenzene (DNCB)-induced dermatitis in mice by reducing excessive chemokine production and inhibiting pro-inflammatory cytokines [93]. In another study, treatment with resveratrol-enriched rice of DNCB-induced AD-like lesions in mice reduced scratching behavior and attenuated the increased secretion of cytokines such as IL-6, IL-31, and IgE [109]. The authors concluded that resveratrol might be used as an alternative therapy to control the chronic skin inflammations seen in AD. Resveratrol also suppressed the immune responses in lipopolysaccharide (LPS)-stimulated RAW 264.7 cells through the SOCS1 pathway. Resveratrol predominantly upregulates the expression of SOCS1 and blocks JAK-STAT signaling [110]. These reports indicate that resveratrol might be developed as a promising therapeutic agent in the treatment of AD.

### 3.2. Leonurine

Leonurine (4-guanidino-n-butyl syringate, Figure 1B) extracted from Chinese motherwort *Herba leonuri* is a natural alkaloid with immense traditional medicinal benefits, including treatment of gynecological, cardiovascular, nervous, cancer, uterine, and skin diseases [111,112,113]. The leaf shoot of *H. leonuri* reduces itching and was used to treat contagious skin diseases such as active shingles in ancient people [114]. Pharmacologically, *H. leonuri* exhibited a protective function against skin photoaging caused by UV-irradiation damage by regulating antioxidative enzymes and the expression of Bax/Bcl-2 to decrease the apoptosis of cells [115]. Further, *H. leonuri* also reduced the acne in a rat ear acne model by the reduction of serum IL-6 levels [116]. Leonurine, one of the active constituents of *H. leonuri*, possesses anti-oxidative, anti-inflammatory, antitumor, antidiabetic, and cardiovascular protective effects [117].

In a previous study, an antitumor role of leonurine was reported, involving inhibition of lung cancer in human non-small-cell lung cancer H292 cells through a mitochondria-dependent pathway [113,118]. Further, leonurine upregulated SOCS5 expression and inhibited JAK/STAT3 signaling, enabling leonurine to inhibit chronic myeloid leukemia malignancy [119]. Leonurine regulates various physiological and pathological mechanisms such as lipid and glucose metabolism, oxidative stress, inflammation, fibrosis, and apoptosis [120]. Based on its multi-mechanistic role, including enhancing SOCS5 expression, leonurine might be further developed as a potential biomolecule in treating AD and other inflammation-mediated skin disorders.

### 3.3. Astragalin

Astragalin (kaempferol-3-*O*-β-d-glucoside, Figure 1C) is a flavonoid present as one of the active constituents in various plant species including Ebenaceae, Rosaceae, and Eucommiaceae families. Astragalin has been shown to have a variety of pharmacological benefits including antidiabetic, anti-osteoporotic, antiulcer, neuroprotective, cardioprotective, anti-obesity, anticancer, anti-inflammatory, and antioxidant properties [121,122]. In cosmeceutical use, astragalin inhibits collagenase activity, thereby preventing wrinkles and controlling pigmentation [123]. Due to its potential ROS scavenging and inflammatory chemokine inhibition, astragalin helps as a photo-protective agent against UV-induced damage to the skin [124].

Astragalin has an inhibitory effect on TNF-α, IL-1β, and IL-6 production in J774A.1 cell lines and LPS-induced inflammatory responses by attenuating the activation of the NF-kB signaling pathway [125,126]. Further, astragalin was found to have a significant anti-AD potential [127,128]. Astragalin from Japanese persimmon extract inhibited the severity of dermatitis, transepidermal water loss, and serum IgE levels and reduced the IL-4 and IL-13 levels of spleen T-cells in NC/Nga AD-model mice [127,128]. Further, astragalin significantly increased SOCS5 expression in ovalbumin (OVA)-induced allergic inflammation in the murine asthma model, as SOCS5 is known to prominently reduce Th2 differentiation by inhibiting IL-4 signaling [129]. Due to the regulation and modulation of various molecular targets involving inflammatory cytokines (SOCS-3, SOCS-5, IL-1β, IL-4, IL-6, IL-8, IL-13, MCP-1, CXCL-1, CXCL-2, and IFN-c), astragalin has immense research potential to be developed as a promising candidate in the treatment of AD.

### 3.4. Diosmetin

Diosmetin (5,7,3′-trihydroxy-4′-methoxyflavone, Figure 1D), also known as 4′-methylluteolin, is a flavonoid occurring naturally from citrus fruits [130]. Pharmacologically, diosmetin possesses anticancer, antimicrobial, antioxidant, estrogenic, and anti-inflammatory activities [130,131,132]. Diosmetin is well documented to enhance antioxidant activity by inhibiting ROS and increasing intracellular antioxidant status. Further, diosmetin reduces nitric oxide production, inhibits the TNF-α levels in macrophages, and suppresses the apoptosis in T48 cells by regulating AKT and ERK protein kinases [133,134]. In a recent study, diosmetin significantly ameliorated inflammatory responses via STAT1/CXCL10 signaling in a high-fat-diet-induced nonalcoholic steatohepatitis (NASH) mouse model and palmitic-acid-stimulated HepG2 cells, indicating diosmetin as a potential biomolecule in suppressing inflammation in NASH conditions [135].

In relation to inflammation-mediated skin disorders, diosmetin and its glycoside diosmin greatly reduced the AD-like lesions in DNCB-induced murine models [136]. It is well documented that overexpression of IL-4 reduces the expression of major proteins in the skin barrier function, including FLG, involucrin, and loricrin [11,134]. Diosmetin improved the AD-like lesions by inhibiting the TEWL and reduced the IgE and IL-4 expression in RBL-2H3 cells and AD mouse models [136]. The authors claimed that diosmetin can effectively cure AD-like pathology by improving skin barrier dysfunction and reducing the severity of dermatitis and other skin inflammatory diseases.

### 3.5. Albaconol

Several bioactive constituents, including triterpenes, lipids, phenols, polysaccharides, and polysaccharopeptides with immense biological properties such as immunomodulatory and antitumor drugs, were isolated from mushrooms [137,138]. *Albatrellus confluens* (Alb. & Schwein.) Kotl. & Pouz, a terrestrial ectomycorrhizal polypore fungus, is an edible species from the family Albatrellaceae. *A. confluens* and its active constituents, including grifolin, neogrifolin, albaconol, and conflamides D and E, are well documented to exhibit various pharmacological and microbiological effects, including anti-oxidant, anti-biotic, anthelmintic, anti-proliferative, antitumor, immunosuppressive, and anti-inflammatory effects [139,140,141,142]. In the cosmeceutical industry, *A. confluens* is a potential ingredient (Evercalm™) used as a humectant, a skin conditioner, and a redness relief serum preventing sensations of itching and discomfort, as well as lessening the feeling of burning, stinging, and tightness on the outermost layer of the skin.

In particular, albaconol (prenylated resorcinol, Figure 1E), a naturally occurring bioactive metabolite, exhibits potential immunomodulatory, immunosuppressive, antitumor, and anti-inflammatory properties and acts as a partial vanillin receptor agonist [141,142]. Albaconol has the ability to influence DNA topoisomerase activity by stimulating DNA cleavage and inhibiting reunion in human tumor cell lines, thereby exhibiting significant antitumor effects [138,143]. Further, albaconol attenuated LPS-stimulated expression of proinflammatory responses, MHC-II expression, and T-cell-stimulating capacity of DCs [142]. Previous studies indicated that macrophages stimulated with LPS induced SOCS1, which plays a downregulating role in protecting the host from fatal responses to LPS [62,144]. Further, SOCS1 overexpression inhibits LPS-induced IκB phosphorylation, resulting in the suppression of NF-κB transcriptional activity [145]. Albaconol induced the upregulation of SOCS1 expression and inhibited NF-κB activation and Th2 differentiation [141]. Data revealed that albaconol exhibits multiple biological activities as a potential immunosuppressive and anti-inflammatory agent and might be developed as a challenging therapeutic target in the treatment of AD.

### 3.6. β-d-Mannuronic Acid

β-d-mannuronic acid (M2000, Figure 1F), a uronic acid monosaccharide, is a component of algenic acid along with guluronic acid, found predominantly in marine brown algae [146]. M2000 is a new member of the group of immunosuppressive agents, with properties of nonsteroidal anti-inflammatory drugs [147]. The therapeutic benefits of M2000 include the treatment of oxidative stress and inflammation-related disorders such as diabetes, aging, neurodegeneration, inflammatory bowel disease, rheumatoid arthritis, tumors, and cardiac disorders [148,149,150,151,152,153,154,155,156]. M2000 showed protective effects against adjuvant-induced arthritis, experimental autoimmune encephalomyelitis, nephrotic syndrome, and acute glomerulonephritis during in vitro and in vivo examinations with maximum tolerance, and its efficacy was proved in various laboratory experimental models [157,158,159]. Further, M2000 showed higher tolerance and biocompatibility in attenuating rat paw edema and joint destruction in a rat arthritis model compared to diclofenac, piroxicam, and dexamethasone [151]. Being a copolymer in alginates, M2000 aids in preventing skin aging and cutaneous disorders due to its potential antioxidant effects and is used in various cosmetic applications [160]. Further, M2000 from brown algae *Padina boryana* derived alginate attenuated the skin damage from particulate-matter-induced inflammation in keratinocytes and fibroblasts via inhibiting NF-κB-associated signals and MAPK pathway molecules [161].

In a recent clinical study, M2000 was reported to regulate the levels of STAT in rheumatoid arthritis (RA) patients, thereby proving its ability in the management of RA [162]. Recent immunopharmacological studies have explored the role of M2000 in SOCS proteins expression in chronic inflammatory disorders [163,164]. M2000 significantly enhanced the expression of SOCS1 and inhibited the production of proinflammatory cytokines in LPS-stimulated HEK293 TLR2 and peripheral blood mononuclear cells, thereby proving its beneficial effects in autoimmune and inflammatory disorders [163]. Further, a clinical study in RA patients indicated that oral administration of M2000 exhibited positive effects in regulating the gene expression of miR-155 and its target molecule SOCS1. SOCS1 was significantly expressed in the patients treated with M2000 compared with control subjects, indicating the role of M2000 in targeting SOCS proteins [164].

In another study, M2000 exhibited significant immunosuppressive and anti-inflammatory properties by upregulating the SOCS1/SOCS3 genes and reducing IL-6 and TNF-α levels in the serum of progressive multiple sclerosis (MS) patients [165]. Further, in a much more recent clinical study by the same group, M2000 also significantly reduced the gene expression of IL-1β, IL-17A, STAT1, and STAT3 in the serum of MS patients, indicating the involvement of M2000 in regulating STAT signaling [166]. Taking the preclinical and clinical data together, the scope emerges for extensive research on M2000 as a potential natural biomolecule in the treatment of various inflammation-mediated skin diseases including AD.

### 3.7. Luteolin

Luteolin (3′,4′,5,7-Tetrahydroxyflavone, Figure 1G), a yellow crystalline flavone isolated from the plant *Reseda luteola* principally for its yellow dye compound, is found in a variety of natural plant sources including leaves, barks, rinds, and pollen. As a dietary source, luteolin can be found in various fruits, vegetables, oils, and seeds such as carrots, apple skins, peppers, celery, olive oil, peppermint, thyme, rosemary, and oregano [167]. According to ethnopharmacological evidence, luteolin has antioxidant, anti-inflammatory, antimicrobial, neuroprotective, and anticancer activities [168]. Luteolin has been well documented to possess significant antioxidant, anti-inflammatory and anti-allergic effects and is beneficial in various diseases including skin psoriasis, gout, and asthma due to regulating multifactorial inflammatory mechanisms such as ROS scavenging and suppressing proinflammatory cytokines and chemokines mediated via NF-κB, AKT, and MAPK pathways [169]. Apart from the regulation of various inflammatory pathways, reports have also revealed that luteolin upregulates the SOCS proteins expression mediated by JAK/STAT signaling and could alter the STAT3/IRF-1, NF-κB, and AP-1 pathways.

Luteolin was found to increase the expression of SOCS3 in LPS- and IFN-γ-stimulated BV-2 cells, thereby inactivating STAT1 signaling and attenuating inflammatory responses [170]. Further, luteolin predominantly suppressed STAT1 and STAT3 phosphorylation in cytokine-induced HT-29 and RAW264.7 cells [171,172,173] and reduced the excessive production of inflammatory synoviocytes in RA patients by regulating the NF-κB- and JAK/STAT-signaling pathways [174].

In relation to skin diseases, topical application of luteolin reduced cutaneous reactions, scratching, and vascular permeability induced by various triggers, including DNCB, histamine, and serotonin, by suppressing the inflammatory responses [175]. Luteolin also suppressed the clinical symptom of AD-like skin damage in mice and controlled the dinitrofluorobenzene (DNFB)-induced mouse ear edemas [176,177]. Luteolin significantly inhibited the IgE levels and reduced the oxidative stress and inflammatory mediators including IL-1β, IL-6, TNF-α, IFN-γ, IL-4, IL-33, IL-8, and IL-17A in murine and canine AD models [178,179]. The ability to be absorbed by and penetrate into the skin make luteolin one of the prominent biomolecules to be used as an anti-phlogistic medicament in skin disorders [180].

Recently, the potential of luteolin in skin aging and inflammation was reviewed, giving insights into the ability of luteolin in the treatment of skin aging, skin cancer, wound healing, psoriasis, contact dermatitis, and AD [181]. The authors indicated that luteolin acts by various mechanistic pathways such as NF-κB, JAK/STAT, and TLR signaling, thereby modulating the inflammatory conditions of the skin. Based on these reports and clinical data, the involvement of luteolin in the SOCS3/JAK2/STAT3-signaling pathway could be a new alternative pharmacological treatment for AD.

### 3.8. Curcumin

Curcumin (1,7-bis(4-hydroxy-3-methoxyphenyl)-1,6-heptadiene-3,5-dione, Figure 1H), one of the major active components of turmeric spice derived from the rhizomes of *Curcuma longa*, is widely used in several Eastern countries for its immense traditional and ethnopharmacological benefits, including anti-inflammatory, antioxidant, neuroprotective, antitumor, antidiabetic, hepatoprotective, and antimicrobial activities [182,183]. Dermatologically, curcumin is well documented to be an effective agent in the treatment of various skin infections and diseases including burns, wounds, surgical scars, photo-damaged skin, psoriasis, and eczema, and it acts as an anti-aging and skin regenerative remedy [184,185,186]. Further, due to curcumin’s safety profile even at higher doses, it is considered to be an attractive natural biomolecule in the management of various skin tumors and is used as a chemoprevention agent in conditions such as scleroderma, psoriasis, and cancers of the skin [185,187,188,189,190].

Earlier reports revealed that curcumin potentially regulated the SOCS proteins expression mediated through JAK/STAT pathways in various experimental studies. Curcumin significantly ameliorated myeloproliferative neoplasms and leukemia in K562 and HEL cells by elevating the expression of SOCS1/SOCS2 and acts as a histone deacetylase (HDAC)8 activity inhibitor [191]. HDAC inhibitors are well reported to attenuate JAK/STAT signaling through the upregulation of SOCS1 and SOCS2 [192,193]. Curcumin also exhibited anti-inflammatory effects in LPS-stimulated RAW 264.7 murine macrophages by preventing the inhibition of SOCS1 and SOCS3 expression, indicating that curcumin is a potential therapeutic in the treatment of infections mediated by chronic inflammatory conditions [194].

In a similar study, curcumin ameliorated the neuroinflammatory responses in LPS-induced BV-2 microglial cells by increasing the expression of SOCS1 and reducing the phosphorylation of JAK2/STAT3 pathways, indicating the potential of curcumin to address neuroinflammation through modulation of JAK/STAT/SOCS signaling [195]. Curcumin also increased anti-inflammatory responses in the management of IBD in an experimental colitis 2, 4, 6-trinitrobenzene sulfonic acid (TNBS)-induced model in mice. Curcumin inhibited the DC costimulatory molecules and inhibited the phosphorylation of JAK2, STAT3, and STAT6. Further, curcumin highly expressed downstream signaling proteins including SOCS1 and SOCS3. The authors concluded that curcumin restored the TNBS-induced imbalance in inflammatory and immunologic responses by attenuating the DC activation through modulation of JAK/STAT/SOCS signaling in colitis mice [196,197].

In a recent study, treatment with curcumin in OVA-induced AD mice significantly attenuated the increased expression of TH2-related cytokines and reduced the activation of STAT6, indicating that curcumin might be beneficial in the treatment of AD and associated infections [198]. In view of the traditional and pharmacological evidence that curcumin is involved in regulating and modulating various inflammatory pathways including JAK/STAT/SOCS signaling, one can confirm curcumin to be a promising therapeutic natural biomolecule in dermatological applications such as treating AD and related proliferative skin infections.

### 3.9. Naringenin

Naringenin ((2*S*)-4′,5,7-trihydroxyflavan-4-one, Figure 1I), a colorless and flavorless flavone, is found in a variety of fruits and herbs including tomatoes and grapes. Naringenin has immense biological effects ranging from antimicrobial, anti-inflammatory, antioxidant, anticancer, antidiabetic, cardioprotective, and neuroprotective effects [199]. In relation to treating allergic immune-related diseases and skin disorders, naringenin has the potential to exhibit antiasthma, anti-aging, and anti-photoaging effects and acts as a skin-protective agent in cosmeceuticals [200,201,202]. Previous reports indicated that naringenin ameliorated LPS-induced skin senescence, UV-irradiated skin inflammation, heat-induced skin damage, and skin fibrosis in various experimental models [202,203,204,205]. In vascular endothelial cells stimulated by cytokines, naringenin effectively induced SOCS3 expression and promotor activity by suppressing IL-6 stimulated STAT-3 phosphorylation [206].

The potential anti-inflammatory, anti-allergic, and anti-oxidant properties exhibited by naringenin make this flavonoid a pertinent biomolecule and a natural remedy in the treatment of AD. Naringenin inhibited DNCB-induced skin lesions and ameliorated skin inflammations in a NC/Nga mice model of AD [207,208]. Naringenin significantly inhibited IFN-γ and IgE levels and reduced the infiltration of mast and T-cells in skin lesions. Further, naringenin suppressed the M1-like macrophage phenotype and inflammatory cytokine protein expression, including NF-κB and extracellular signal-regulated kinase pathways, in AD mice [207,208]. Based on these reports, further research on naringenin might hold promise for the development of naringenin as an important potential biomolecule in the treatment of inflammatory skin disorders such as AD.

### 3.10. Quercetin

Quercetin (3,3′,4′,5,7-Pentahydroxyflavone, Figure 1J), one of the abundant dietary flavonoids occurring naturally, is found in several fruits and vegetables and has a long history of medicinal usage with significant efficacy and no side effects [209]. Pharmacologically, quercetin is well reported to possess antioxidant, anti-aging, and anti-inflammatory effects [210]. Quercetin is also well documented as a potential anti-inflammatory molecule in the treatment of allergic disorders including asthma, rhinitis, and AD [211].

In relation to AD, quercetin exhibited anti-AD effects by activation of nuclear factor erythroid 2-related factor 2 (Nrf2) and heme oxygenase (HO-1) pathways [212]. Further, quercetin strongly inhibited ROS and protected keratinocytes against oxidative damage [213]. Furthermore, quercetin suppressed Th2-related cytokine expression, including TSLP and thymus and activation-regulated chemokine (TARC), in an AD-like Nc/Nga mouse model and tetradecanoylphorbol 13-acetate (TPA)-induced skin inflammation [214,215]. In addition, quercetin played a major role in suppressing the abnormal activation of JAK-STAT signaling and inhibited the overexpression of IL-4, 5, and 13 in inflammatory diseases. However, one study found that quercetin had anti-proliferative effects of IFN-α on cancer cell proliferation due to activating JAK/STAT pathway signaling by inhibiting SHP2 but had no effect on SOCS expression, urging more research into the effect of quercetin on SOCS proteins in the treatment of AD and other skin inflammation [216].

## 4. Conclusions and Future Perspectives

AD is a common inflammatory skin disease, with approximately 230 million people suffering worldwide. A significant number of experimental studies on various JAK/STAT pathway inhibitors including tofacitinib, ruxolitinib, baricitinib, upadacitinib, abrocitinib, cerdulatinib, gusacitinib, and delgocitinib have been reported and/or are currently under investigation for the evaluation of their clinical use in AD [217,218,219]. Further, therapeutic trials are currently underway both in clinical and pre-clinical testing using SOCS anti-sense oligonucleotides, shRNA, and peptide mimetics as simple negative-feedback regulators for fine-tuning immune response and inflammation in various disorders including AD [220,221]. However, their long-term safety profile and adverse therapeutic effects, including increased risks of infection, malignancies, serum lipids, and the development of certain cytopenias, were not elaborately addressed. Further, the high cost involved in handling the available synthetic JAK/STAT inhibitors and peptide molecules and their treatment regimens should be simultaneously considered. Therefore, complementary and alternative medicinal therapies involving various natural products and their derived biomolecules for enhancing SOCS expression or mimicking its consequences and regulating JAK/STAT signaling might be appropriate as treatment options in AD therapy.

Several natural products and their derived small molecules with immunosuppressive and anti-inflammatory effects are being widely used in the treatment of inflammation-mediated skin infections including AD. The highly conserved negative regulation of JAK/STAT signaling has emerged as a challenging mechanistic approach, as it is involved in various disease pathways [40,222,223]. Further, naturally derived biomolecules such as oleanolic acid, cathechins, artemisinins, emodin, capsaicin, hesperidin, 5,7,3′-triacetyl hesperetin, and silibinin have achieved significant importance and can influence and modulate JAK/STAT signaling. However, a detailed study on individual agents as potential biomolecules in AD pathogenesis through targeting JAK/STAT/SOCS-associated signaling is quite essential. In this review, the prominent natural-product-derived biomolecules exhibiting positive influence on SOCS1, SOCS3, and SOCS5 expression as negative regulators of the JAK/STAT pathway in controlling inflammatory infections including AD were discussed. A summary of the natural biomolecules reviewed with experimental evidence and target actions, including their dose range, is shown in Table 1.

**Figure 2:**
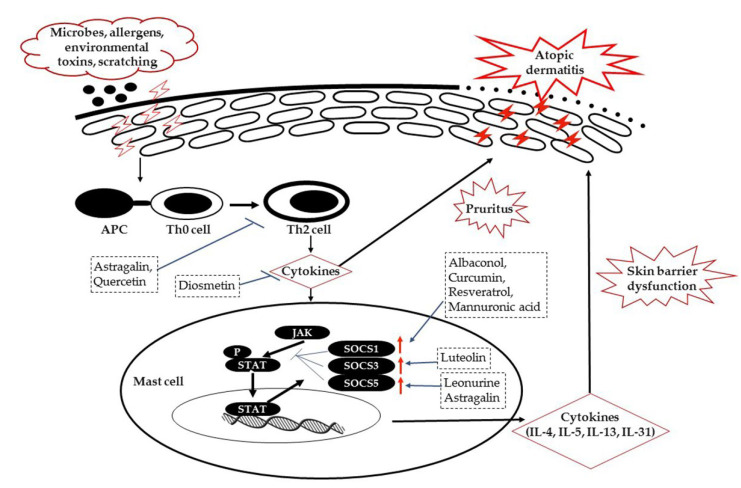
Schematic diagram of selected natural biomolecules and their possible target sites in JAK/STAT/SOCS signaling in AD pathogenesis. Pathogenic microbes, allergens, and environmental toxins often attack the skin, accelerating inflammation by releasing cytokines, which are activated by antigen presenting cells, leading to AD. Antigen-presenting cells activate naïve Th0 cells, making them mature and differentiate into Th2 cells. TH2 cells release cytokines, leading to activation of the JAK/STAT pathway in stimulated mast cells and further enhancing the release of inflammatory cytokines, which are causative factors for various allergic diseases including AD. The cytokines released cause pruritus and skin barrier dysfunction, thereby increasing the severity of AD. Simultaneously, the negative regulators of JAK/STAT signaling, namely SOCS proteins (SOCS1, 3, and 5), are activated to block STAT phosphorylation to control excessive cytokine-mediated skin damage. Possible involvement of selected natural biomolecules at target sites through inhibiting the cytokine release via Th2 cells or enhancing the expression of SOCS proteins, thereby negatively regulating activation of JAK/STAT signaling in the management of AD pathogenesis, is shown.

In our literature survey, the majority of the studies reported a lack of direct and well-established research findings concerning ameliorating AD pathogenesis by targeting JAK/STAT/SOCS signaling. However, the data had scientific importance for future directions in exploiting the possible beneficial role of new and existing natural biomolecules in AD management. Further, it is to be noted that none of the natural biomolecules have entered the drug market or clinical phase for their pharmacological efficacy and safety as potential agents in AD management through targeting JAK/STAT/SOCS signaling, posing a challenge for scientists and clinicians for further research. A proposed schematic diagram illustrating the possible target sites aimed at targeting the cytokines and JAK/STAT/SOCS-signaling pathway is shown in Figure 2.

JAK/STAT/SOCS signaling plays one of the major roles in immune cells such as mast cells, T-helper cells, and macrophages, which are involved in the pathogenesis of AD. AD is a Th2-dominant inflammatory skin disorder, and the JAK/STAT pathway is well-known for its role in inflammatory and immune regulation. The Th2 cytokines, especially IL-31, induce pruritus in AD and activate the JAK1/JAK2 and STAT3 pathways. SOCS1 and SOCS5 are major inhibitors of JAK phosphorylation and are involved in regulating Th2 differentiation by inhibition of IL-4. SOCS3 also inhibits JAK phosphorylation, but it upregulates Th2 differentiation via suppression of Th1 development. Understanding the anti-inflammatory mechanisms of JAK/STAT/SOCS signaling and the immense role played by natural biomolecules in regulating inflammatory mechanisms might pave the way to the development of novel therapies with tremendous advancements in the management of moderate to severe AD pathogenesis. Further, thorough pre-clinical and clinical investigations into natural biomolecules regulating JAK/STAT/SOCS signaling might help shed light on other inflammation-related human disease management strategies for diseases such as allergies, asthma, arthritis, and cancers.

Active biomolecules from natural products, including flavonoids, terpenoids, glycosides, alkaloids, and polysaccharides, have proven beneficial for their effectiveness and safety in the treatment of AD. Future research should focus on elaborate studies involving various in vitro and animal models on attenuating AD pathogenesis through targeting JAK/STAT/SOCS signaling. The multifaceted signal transduction pathways exerted, the consequences of using these natural biomolecules as SOCS protein mimetics or endogenous enhancers in clinical AD settings, and their outcomes warrant further research. Further studies should also focus on understanding whether the involvement of natural biomolecules in enhancing the expression of SOCS proteins is either through directly binding to the SOCS protein moieties or through transcriptionally regulating the SOCS gene expression. In view of the available literature, modulation of JAK/STAT/SOCS-signaling pathways using natural biomolecules could represent a potential therapeutic target for drug development aimed to counteract inflammation-mediated AD and other skin infections.

## Figures and Tables

**Table 1:** Selected natural biomolecules targeting cytokines and JAK/STAT/SOCS signaling and their possible role in treating AD and other inflammatory disorders.

Natural Compound	Source	Experimental Evidence	Dosage	Target Action	Ref.
Resveratrol	Resveratrol-enriched rice	DNCB-induced AD NC/Nga mice	2.5% *w*/*v*	↓ scratching behavior, ↓ dermatitis, ↑ skin hydration, ↓ IL-6, IL-1β, IgE, IL-31	[93,109]
		TNF-α/IFN-γ-stimulated HaCaT cells	1–50 µg/mL	↓ IL-6, IL-31, IgE	
	Grapes, Berries, *Polygonum cuspidatum*	LPS-stimulated RAW 264.7 cells	0–20 µM	↓ inflammatory cytokines, ↓ STAT1/STAT3, ↑ SOCS1	[107]
Leonurine	*Herba leonuri*	CML cells	0.4 and 0.6 mM	↑ SOCS5, ↓ JAK2/STAT3 pathway	[119]
		Xenograft BALB/c animal model	150 mg/kg/4 weeks		
Astragalin	Persimmon, *Rosa agrestis* leaves	OVA-induced BALB/c asthma animal model	1 mg/kg/day	↓ IL-4, IL-5, IL-13, IgE, ↑ SOCS5, ┴ Th2 differentiation	[129]
Diosmetin	Citrus fruits	RBL-2H3 cells	5, 10, 25 mg/kg/day	↓ IL-4	[136]
		DNCB-induced AD mice	50 mg/kg	↓ IL-4 and IgE, ┴ Th2 differentiation	
Albaconol	*Albatrellus confluens*	LPS-activated RAW 264.7 cells	5 µg/mL	↑ SOCS1, ┴ IL-6, IL-1β, NF-κB	[141]
β-d-Mannuronic acid	Marine brown algae	RA patients	500 mg/day	↑ SOCS1, ┴ IL-6 and TNF-α	[164]
		MC patients	-	↑ SOCS1 and SOCS3, ↓ IL-1β, IL-17A, STAT1, and STAT3	[166]
Luteolin	*Reseda luteola*, Apple skin, Rosemary, Oregano, Peppermint	LPS & IFNγ-stimulated BV-2 cells	20 µM	↑ SOCS3, ┴ STAT1	[170]
		In vitro canine AD model	1–8 µM	↓ IL-33, IL 1β, IL-6, IL-8	[178]
		DNCB-induced murine AD model	10, 20, and 30 mg/kg	↓ IgE, IL-1β, IL-6, TNF-α, IFNγ, IL-4, IL-17A, NF-κB	[179]
Curcumin	*Curcuma longa*	LPS-stimulated BV-2	10, 30, and 50 μM	↑ SOCS1, ↓ JAK2/STAT3	[195]
		TNBS-induced colitis mice	100 mg/kg/wk	↑ SOCS1,SOCS3, ┴ JAK2/STAT3, STAT6	[196,197]
		OVA-induced murine AD model	20 mg/kg/wk	↓ Th2 expression, ↓ IL-4/IL-5/IL-13/IL-31, ↓ STAT6	[198]
Naringenin	Tomato and grapes	DNFB-induced NC/Nga mouse AD model	50 and 100 mg/kg	↓ IgE, IFN-γ, ↓ Infiltration of mast and T-cells	[207]
		IL-6-induced HUVEC cells	100 µM	↑ SOCS3, ↓ STAT3	[206]
Quercetin	All fruits and vegetables	House-dust-mite-allergens-induced AD in Nc/Nga mice	2.5 mM	↓ neoangiogenesis	[214]
		TNF-α-stimulated HaCaT cells	1, 5 and 10 µM	↓ Th2 cytokine expression, TSLP, TARC	
		IFNα-induced HepG2 and Huh7 cells	1–10 μM	┴ SHP2, ↓ JAK1/STAT3, no effect on SOCS1/SOCS3	[216]

**Abbreviations:** AD: atopic dermatitis; DNCB: 2,4-dinitrochlorobenzene; IL: interleukin; IgE: immunoglobulin; TNF-α: tumor necrosis factor-alpha; IFN-γ: interferon gamma; LPS: lipopolysaccharide; STAT: signal transducer and activator of transcription; SOCS: suppressor of cytokine signaling; CML: chronic myeloid leukemia; JAK: janus kinase; OVA: ovalbumin; Th2: T-helper type 2; NF-κB: nuclear factor kappa-light-chain-enhancer of activated B; RA: rheumatoid arthritis; MC: multiple sclerosis; TNBS: 2,4,6-trinitrobenzene sulfonic acid; DNFB: 2,4-dinitrofluorobenzene, SHP2: Src homology domain 2 containing tyrosine phosphatase 2; ↑: upregulation; ↓: downregulation; ┴: inhibition.

## Data Availability

Not applicable.
